# Endothelial glycocalyx as a critical signalling platform integrating the extracellular haemodynamic forces and chemical signalling

**DOI:** 10.1111/jcmm.13081

**Published:** 2017-02-17

**Authors:** Ye Zeng

**Affiliations:** ^1^ Institute of Biomedical Engineering School of Preclinical and Forensic Medicine Sichuan University Chengdu China

**Keywords:** glycocalyx, sphingosine‐1‐phosphate, shear stress

## Abstract

The glycocalyx covers the human mammalian cells and plays important roles in stroke, inflammation and atherosclerosis. It has also been shown to be involved in endothelial mechanotransduction of shear stress. Shear stress induces the remodelling of the major component of the glycocalyx including glypican‐1, a cell membrane heparan sulphate proteoglycan. Other factors, such as sphingosine‐1‐phosphate (S1P), protect the glycocalyx against syndecan‐1 ectodomain shedding and induce the synthesis of heparan sulphate. In this study, we reviewed the role of shear stress and S1P in glycocalyx remodelling and revealed that the glycocalyx is a critical signalling platform, integrating the extracellular haemodynamic forces and chemical signalling, such as S1P, for determining the fate of endothelial cells and vascular diseases. This review integrated our current understanding of the structure and function of the glycocalyx and provided new insight into the role of the glycocalyx that might be helpful for investigating the underlying biological mechanisms in certain human diseases, such as atherosclerosis.

## Introduction

The glycocalyx mediates the endothelial mechanotransduction of shear stress and serves as a selective permeability, anti‐inflammatory and anti‐adhesive barrier at the luminal side of the endothelium 1, 2, 3, showing a protective effect on vascular functions. Endothelial cell (EC) injury in atherosclerosis (AS) causes reduction in the endothelial glycocalyx 4, 5. AS is the major pathological basis causing cardiovascular and cerebrovascular diseases. Thrombosis and atherosclerotic plaque rupture lead to acute coronary syndromes including unstable angina, acute myocardial infarction and heart attack 6, 7.

During AS, the changes in structure and function of ECs adapt to the local mechanical (such as shear stress) and chemical (such as vasoactive mediators and cytokines) microenvironments in vessels, which involve various cell activities, such as phenotypic conversion of cells 8 and remodelling of the extracellular matrix and endothelial glycocalyx 9, 10, 11, 12, 13. It is well known that different flow patterns and associated shear stresses are produced with the development of AS 14. Recently, it was reported that shear stress induces clustering of the major components of the endothelial glycocalyx including glypican‐1, a cell membrane heparan sulphate proteoglycan (HSPG) 9, 10.

Sphingosine‐1‐phosphate (S1P) is a lipid mediator produced by sphingolipid metabolism and mostly present in plasma that induces various cellular effects, including proliferation, differentiation, survival and migration 15. S1P is also emerging as a potent modulator of endothelial barrier function and vascular tone 16. The structure of the glycocalyx is also modulated by S1P, which protects the endothelial glycocalyx against shedding and induces its synthesis 9, 10. Thus, both shear stress and S1P play critical roles in modulating the structure and function of the glycocalyx and contribute to vascular homeostasis and remodelling.

This study reviews the research progress on the structure and function of the endothelial glycocalyx, their regulation by shear stress and S1P, and the roles of shear stress and S1P in AS. Finally, we conclude that the glycocalyx is a critical signalling platform for deciding the fate of ECs and vascular diseases, which may be helpful for elucidating the complicated pathological mechanism of AS.

## Research progresses on the structure and function of the endothelial glycocalyx

### Structure of the endothelial glycocalyx

**Figure 1:**
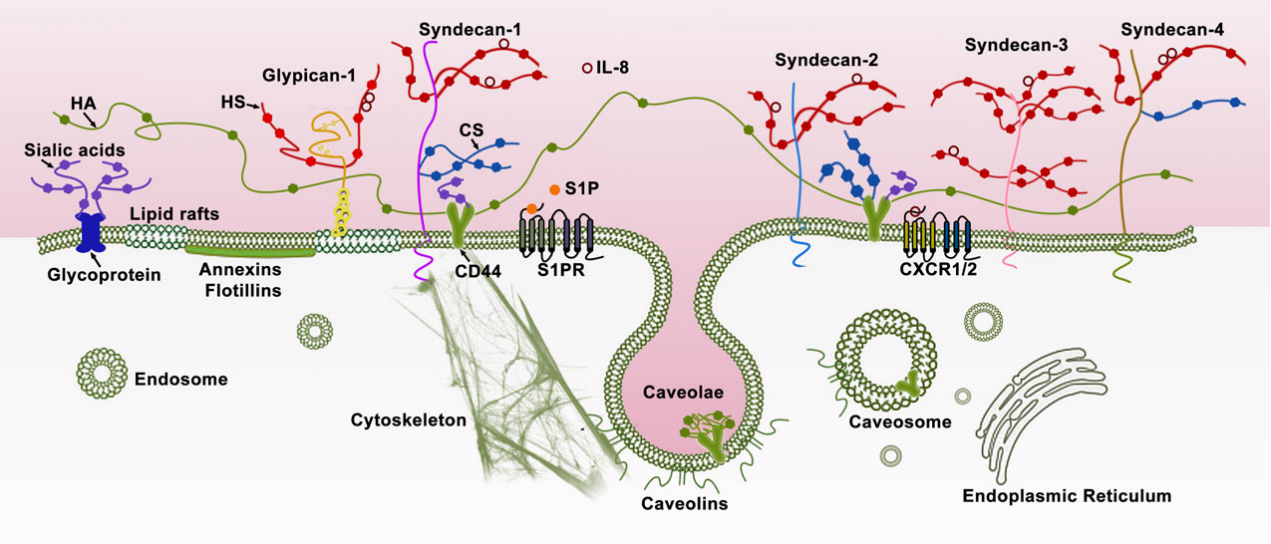
Structure of the endothelial glycocalyx. The endothelial glycocalyx is located at the luminal side of vascular endothelial cells and its soluble components are directly in contact with the bloodstream. The endothelial glycocalyx is mainly composed of glycoproteins bearing acidic oligosaccharides and terminal sialic acids (SA), proteoglycans (PG), such as heparan sulphate proteoglycans (HSPGs; syndecan family and glypican‐1), and glycosaminoglycan (GAG) side chains. The predominant GAGs in endothelial cells are heparan sulphate (HS), chondroitin sulphate (CS) and hyaluronic acid (hyaluronan, HA). HS and CS are attached to PGs. HA binds with receptor CD44. Syndecans (including syndecan‐1, syndecan‐2, syndecan‐3 and syndecan‐4) are single transmembrane domain proteins. Glypican‐1 is an extracellular glycosylphosphatidylinositol (GPI)‐anchored protein, which is localized in lipid rafts. Syndecan‐1 and CD44 interact with the cytoskeleton.

The endothelial glycocalyx lines the luminal side of the vascular ECs and its soluble components exist in a dynamic equilibrium with the bloodstream. The structure of the endothelial glycocalyx has been investigated in depth 12, 17. It is mainly composed of glycoproteins bearing acidic oligosaccharides and terminal sialic acids (SA), proteoglycans (PG) like HSPG (including syndecans and glypican‐1 core proteins) and glycosaminoglycan (GAG) side chains (Fig. 1). The predominant GAGs in ECs are heparan sulphate (HS; >50% of the total GAG pool), chondroitin sulphate (CS) and hyaluronic acid (hyaluronan, HA). HS and CS are attached to PGs, whereas HA does not link to the PG core protein. HA is a kind of non‐sulphating GAG, which binds with receptor CD44.

The HSPG syndecan family has four members: syndecan‐1, syndecan‐2, syndecan‐3 and syndecan‐4. Syndecan‐1 contains five potential GAG attachment sites, three near its NH_2_‐terminal ectodomain and two adjacent to the transmembrane domain near its COOH terminus. CS is only found near the COOH terminus of syndecan‐1 18. Syndecan‐3 contains eight potential GAG attachment sites, five near its NH_2_‐terminal ectodomain and three adjacent to the transmembrane domain near its COOH terminus. Both syndecan‐2 and syndecan‐4 contain three potential GAG attachment sites near their NH_2_‐terminal ectodomain 19, 20. Syndecan‐4 can also contain CS 21. In the HSPG glypican family, only glypican‐1 is expressed in ECs. Glypican‐1 is an extracellular glycosylphosphatidylinositol (GPI)‐anchored protein, which only binds with HS.

In resting conditions, syndecans and glypican‐1 mRNAs in human umbilical vein endothelial cells (HUVECs) are expressed in the order: syndecan‐1>syndecan‐4>syndecan‐3>syndecan‐2>glypican‐1 22. The endothelial glycocalyx is modified under several conditions including disturbed flow exposure in large vessels 23, protease degradation 23, 24, 25, and removal of plasma components, particularly albumin 26.

### Function of the endothelial glycocalyx

The endothelial glycocalyx has various functions 5, 17. First, the negatively charged glycocalyx layer forms an electrostatic barrier for plasma cells and proteins, like albumin. Second, the glycocalyx layer also forms an anticoagulation barrier. Increased serum levels of syndecan‐1 are associated with acute coagulopathy following trauma 27.

The dominant mechanism that defines widespread endothelial dysfunction is impaired expression of constitutive endothelial nitric oxide synthase (eNOS) and production of nitric oxide (NO) 28. Knockdown of glypican‐1 inhibits the activation of eNOS under shear stress 29.

Syndecan‐1, syndecan‐2 and syndecan‐3 might contribute to angiogenesis. Syndecan‐1 plays important roles in EC survival, proliferation and organization into capillary‐like structures 30. Shed syndecan‐2 regulates angiogenesis by inhibiting EC migration *via* CD148 (PTPRJ) signalling 31.

Both HS‐ligand binding and interactions of the PG core protein with cytoskeletal and/or signalling molecules are required for cell adhesion and migration. Depletion of syndecan‐1 29 or syndecan‐4 32 has been shown to cause a failure to sense flow direction and inhibition of flow‐induced alignment *in vitro*. A recent study demonstrated that HS is essential for interleukin (IL)‐8‐induced cell migration 33. After enzymatic removal of HS, we observed significant suppression of the IL‐8‐up‐regulated Rho GTPases including Cdc42, Rac1 and RhoA, IL‐8‐increased Rac1/Rho activity, as well as IL‐8‐induced polymerization and polarization of actin cytoskeleton and an increase in stress fibres.

In cell recruitment, it has been thought that both chemokine oligomerization and binding to GAGs are required. Also, their interactions with GAGs facilitate the formation of the chemokine gradients, which provide directional cues for migrating cells 34. Thus, the glycocalyx could be a good platform to integrate various signals.

## Shear stress induces remodelling of the endothelial glycocalyx

It was well known that a dysfunctional endothelium in the AS‐susceptible location is an early manifestation of AS 35. Vascular endothelial injury in the AS‐susceptible location was the prerequisite for AS formation, while the atherosclerotic plaque was the consequence of subsequent vascular repair induced by shear stress 36, 37, 38.

In the AS‐susceptible location, such as branches, bifurcation and curvatures (*e.g*. the aortic arch) of the arterial tree, the blood stream undergoes tremendous interference and the flow departs from pulsatile, unidirectional shear stress to create flow separation zones that include flow reversal, oscillatory shear stress and sometimes turbulence (chaotic flow) 28, 39. In contrast, flow in adjacent undisturbed flow regions of the arteries is pulsatile and has well‐defined directions. It can be speculated that low shear stress induces the initial lesion, and high shear stress promotes the formation of vulnerable plaques. In the vascular lesion location, a continuous exposure of EC to high shear stress induced an abnormal NO production 40, which might be associated with degradation of the glycocalyx 41 and extracellular matrix through matrix metalloproteinases (MMPs), as well as inflammation.

In a recent study, we detected that transcriptional expression of HSPGs (syndecan family and glypican‐1) in HUVECs responded to the distinct magnitudes of shear stress 22. During the initial 0.5 hr of exposure, syndecan‐1 mRNA was the most up‐regulated, by 4 dyn/cm^2^ of shear stress, and syndecan‐4 mRNA was significantly up‐regulated, by 10 and 15 dyn/cm^2^. After 24 hrs of exposure, the greatest increased HSPG mRNA was syndecan‐4 under 4 dyn/cm^2^, and syndecan‐3 under 15 dyn/cm^2^. These molecular changes that may be associated with vascular homeostasis and endothelial dysfunction revealed the potential candidate components of the glycocalyx in response to cardiovascular diseases.

The glycocalyx plays an important role in EC mechanotransduction of shear stress. Weinbaum *et al*. 42 pointed out that the existence of the endothelial glycocalyx could weaken the shear stress on the vascular EC surface to a negligible level by theoretical analysis. Thi *et al*. 43 further proved that the endothelial glycocalyx is required for the EC cytoskeleton to respond to shear stress. Furthermore, selective degradation of some specific components (such as HS) of the endothelial glycocalyx or silence of specific genes (such as glypican‐1) can inhibit the shear stress‐induced activation of eNOS 29 and the production of NO in ECs 44.

Using confocal microscopy, we discovered that 15 dyn/cm^2^ shear stress induces remodelling of the endothelial glycocalyx 9, 10. At an initial 30 min., 15 dyn/cm^2^ shear stress induced the junctional clustering of HS *via* mobility of GPI‐anchored glypican‐1 in lipid rafts (rapid change). After 24 hrs, 15 dyn/cm^2^ shear stress induced the recovery of HS (adaptive remodelling), which shows a similar distribution to that present in the aorta of rats and mice *in vivo* 45. The increases in syndecan‐3 and syndecan‐4 and glypican‐1 might contribute to the adaptive remodelling of the glycocalyx 22.

The remodelling of the glycocalyx might be associated with changes in various EC functions, such as proliferation, migration, adhesion, eNOS activation and NO production. Degradation of HS has been found to significantly inhibit the motility and proliferative responses of EC to shear stress 46 and greatly enhance the adhesion of leucocytes to the endothelium 47. The NO production increased significantly within minutes under 15 dyn/cm^2^ shear stress 9. Removal of glypican‐1 inhibited the 15 dyn/cm^2^ shear stress‐induced activation of eNOS and further reduced the 4 dyn/cm^2^‐inhibited eNOS activity 41. The glycocalyx damage might switch the role of high shear stress from protecting vessels to accelerating the rupture of an AS plaque. Thus, the glycocalyx could be a good platform to integrate the signals (*i.e*. chemokine and shear stress) that slow or prevent the development of AS by structure remodelling. Once the platform is impaired, the interplay of signals, as well as the involved mechanism might change.

## S1P maintains the integrity of the endothelial glycocalyx

S1P is emerging as a potent modulator of endothelial function in response to injury 16. S1P exerts a variety of biological actions through binding with the specific G protein‐coupled receptor (S1P_1‐5_) on the cell surface to activate signalling cascades or serve as a second messenger 48. Receptor S1P_1‐3_ prevails among all kinds of tissues in the cardiovascular system 49, 50 and has been widely investigated. S1P and its receptor, S1P_1_, was required for embryonic angiogenesis and vascular stabilization 50. S1P can promote the formation of an actin ring around the vascular ECs and strengthen the cell–cell and cell–matrix interactions through S1P_1_, maintaining the permeability of the vascular wall 51. The specific agonist of S1P_1_ significantly inhibits the formation and development of AS but does not influence the S1P level in plasma 52. When mice were fed a high‐fat diet, abnormal vascular phenotype and development of plaque were obvious in the descending aorta in the Apoe^−/−^ and EC‐specific S1PR_1_ null mice (S1PR_1_^f/f^ VE‐cadherin‐Cre‐ER^T2^), but was not evident in the Apoe^−/−^ and S1PR_1_ wild‐type mice 53, showing S1P could maintain the vascular homeostasis and prevent the development of AS through S1PR_1_.

The glycocalyx was seen to be modified after removal of plasma components, particularly albumin 26. It was demonstrated that albumin‐bound S1P inhibits shedding of the syndecan‐1 ectodomain *via* activation of the S1P_1_ receptor in ECs 13 and, thus, maintains the normal vascular permeability in intact microvessels 54. In ECs depleted of plasma protein in a culture medium, the shedding of syndecan‐1 through MMP‐mediated proteolytic cleavage close to the plasma membrane on the external face was also recently demonstrated 13. The shedding of syndecan‐1 ectodomain also removes the attached HS and CS 13.

After complete shedding of the glycocalyx components (including syndecan‐1 with attached HS and CS) by depletion of plasma protein in a culture medium, the addition of S1P induced the recovery of the endothelial glycocalyx *via* the PI3K pathway 11, suggesting the synthesis of glycocalyx also contributes to the integrity of the endothelial barrier. Therefore, it can be concluded that S1P maintains the stability of the glycocalyx through collectively inhibiting the shedding and promoting the synthesis of the glycocalyx, thereby contributing to the maintenance of normal vascular permeability 54, and controlling the cardiovascular and immune functions 51. It is interesting to further investigate the exact intracellular signalling pathway involved in the S1P‐preserved/induced glycocalyx.

## The glycocalyx is a signalling platform for integrating haemodynamic force and sphingosine‐1‐phosphate

Evidence shows the S1P receptor is associated with haemodynamic forces. It was demonstrated that ECs responded to haemodynamics *via* S1P_1_ *in vivo* 53. The vascular endothelial S1P_1_ receptor can respond to flow, transducing the signals into cellular chemical signalling to promote the stabilization of a newly formed vascular network 53. Knockout of S1P_1_ gene in mice manifests as injured vessel maturation and embryonic mortality. The change in S1P_1_ might further induce the remodelling of the glycocalyx.

**Figure 2:**
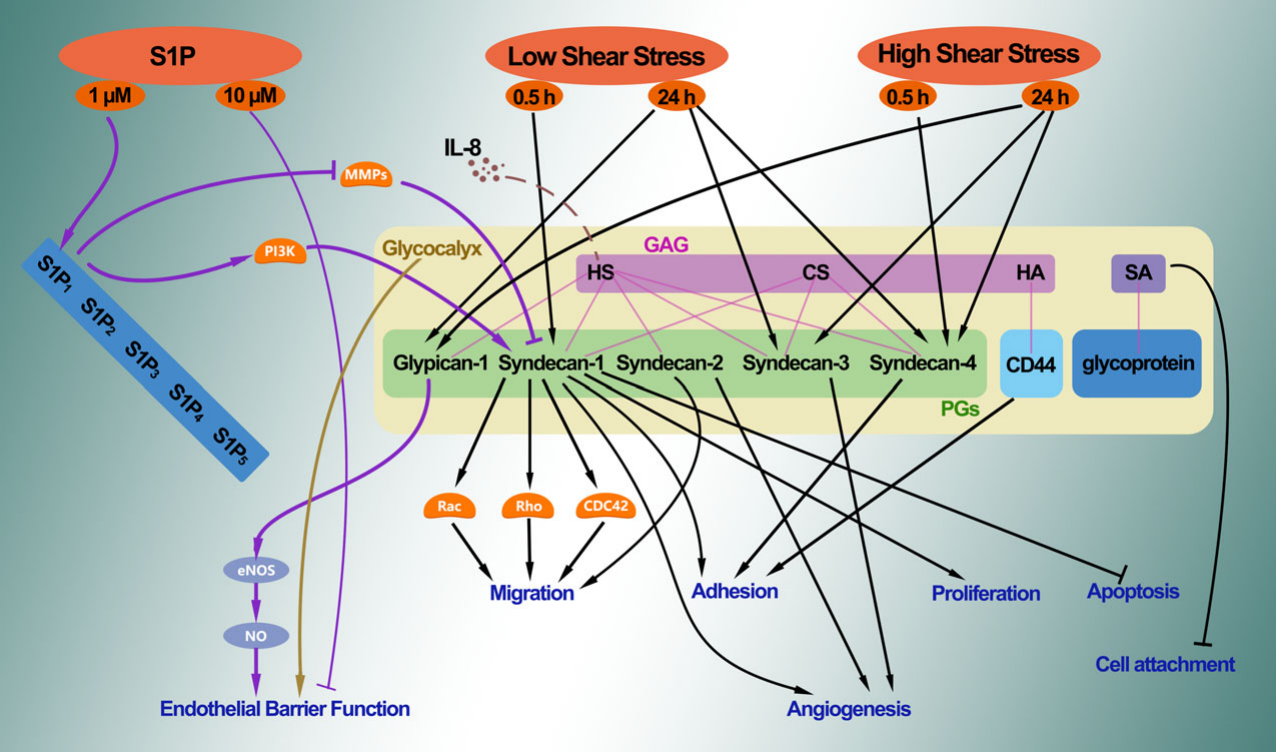
The glycocalyx acts as a signalling platform. Different signalling pathways involved in cellular function, in conditions with or without the glycocalyx. The glycocalyx acts as a signalling platform. Once the platform is destructed, the interplay among these factors and the underlying signalling pathways might be changed.

The glycocalyx is a critical signalling platform that integrates the S1P, shear stress, chemokines and cytokines for maintaining vascular homeostasis (Fig. 2). Once the platform is destructed, the interplay among these factors and the underlying signalling pathways might be changed, which further contributes to the endothelial dysfunction and the development of AS.

To elucidate the pivotal role of the glycocalyx under shear stress and its associated mechanism, a new field—mechanoglycobiology is gradually emerging. Importantly, investigations into the mechanoglycobiological mechanism underlying the remodelling of the glycocalyx could bridge the effects of shear stress, S1P, chemokines and cytokines in AS. We expect that innovations in the mechanoglycobiology field will provide new insight into developing novel prevention and treatment strategies for human diseases.

## Conflict of interest

None declared.
